# A Review of Starch Biosynthesis in Relation to the Building Block-Backbone Model

**DOI:** 10.3390/ijms21197011

**Published:** 2020-09-23

**Authors:** Ian J. Tetlow, Eric Bertoft

**Affiliations:** 1 Department of Molecular and Cellular Biology, College of Biological Science, University of Guelph, Guelph, ON N1G 2W1, Canada; 2 Bertoft Solutions, 20960 Turku, Finland; eric.bertoft@abo.fi

**Keywords:** amylopectin, amyloplasts, amylose, building block-backbone model, cluster model, debranching enzymes, disproportionating enzyme, endosperm, granule morphology, hilum, malto-oligosaccharides, starch, starch synthase, starch branching enzyme, starch phosphorylase, starch structure models, starch synthesis

## Abstract

Starch is a water-insoluble polymer of glucose synthesized as discrete granules inside the stroma of plastids in plant cells. Starch reserves provide a source of carbohydrate for immediate growth and development, and act as long term carbon stores in endosperms and seed tissues for growth of the next generation, making starch of huge agricultural importance. The starch granule has a highly complex hierarchical structure arising from the combined actions of a large array of enzymes as well as physicochemical self-assembly mechanisms. Understanding the precise nature of granule architecture, and how both biological and abiotic factors determine this structure is of both fundamental and practical importance. This review outlines current knowledge of granule architecture and the starch biosynthesis pathway in relation to the *building block-backbone* model of starch structure. We highlight the gaps in our knowledge in relation to our understanding of the structure and synthesis of starch, and argue that the building block-backbone model takes accurate account of both structural and biochemical data.

## 1. Introduction

Since the discovery around 1940 that starch is composed of the two polysaccharides amylose and amylopectin, the molecular branched structure of the latter has been a subject of intense research. In 1940, Meyer and Bernfeld [1] suggested that amylopectin forms a randomly branched dendritic structure. Later, Gunja-Smith et al. [2] found that the α-glucan chains of amylopectin has a bimodal size-distribution, as opposed to the monomodal size-distribution of chains in glycogen, and thus likely has a different structure from glycogen [3]. Nikuni [4], French [5], and Robin et al. [6] suggested a cluster model for amylopectin, which later was refined by Hizukuri [7] based on a polymodal size-distribution of the amylopectin chains, and this model has been largely accepted by the research community. However, more recent experimental data has challenged the accuracy of the cluster model [8,9].

This review attempts to reconcile current knowledge of starch structure with our evolving view of the complex biochemical pathway responsible for its synthesis in relation to the *building block backbone* model proposed by Bertoft [10]. An overview of the structural components of the starch granule, amylose and amylopectin are presented in relation to granule architecture and their relationship to the backbone model. This is followed by an outline of the starch biosynthesis pathway occurring in plastids, with an attempt at integration of biological activities within the context of the backbone model. It will become apparent that current knowledge does not yet extend to explaining how the higher-order elements of granule architecture arise from the various enzyme activities responsible for the synthesis of building blocks.

## 2. Components of Starch Granules

Starch granules are deposited inside the plastids of higher plant cells as discrete, semi-crystalline granules that to the most part consist of the two polyglucans amylose and amylopectin, of which the latter constitutes the major component, and importantly, the component which defines the fine structure of the granule [8].

### 2.1. Amylopectin

Amylopectin is a highly branched macromolecule with huge molecular weight in the order of 10^7^–10^8^ Dalton. It consists of a large number of comparatively short α-glucan chains having a degree of polymerization (DP) of approximately 18 to 25 glucosyl units interconnected by α-(1,4)-d-glycosidic linkages. The chains are linked to each other through branches of α-(1,6) linkages, which comprise about 5% of all glucosyl linkages in most starches. The frequency and clustered positioning of the α-(1,6) linkages is thought to be a crucial determinant of the water-insoluble nature of starch, as opposed to water-soluble glycogen with more evenly distributed α-(1,6) linkages in the order of 9% [11,12]. Given the importance of amylopectin in determining structural and functional properties of starch granules a major focus of the following review is devoted to the structural organization and synthesis of amylopectin.

### 2.2. Amylose

The fact that all natural starch granules contain a significant, but minor, proportion of amylose indicates that it plays an important structural role and must impart a degree of fitness for the plant during growth and development. Amylose is the minor polyglucan component of starch and generally comprises approximately 15 to 35% by weight of the carbohydrate content in most starches, although there is considerable variation in this figure [13]. In some plant species amylose content may be as low as 5 to 8% (e.g., Arabidopsis (*Arabidopsis thaliana* (L.) Heynh), and in so-called *waxy* starches (a genetic mutation, see later) the amylose content is very low—or may even be completely absent—and in so-called high-amylose starches the content might be much higher, reaching close to 100% [14]. The precise biological role of amylose is not understood. It has been suggested to enhance storage capacity and packing of the starch granule, but analysis of normal and *waxy* starches indicate no discernible differences in polymer density [15]. The α-glucan chains in amylose are much longer than in amylopectin and have typically a few hundred to thousand α-(1,4)-linked d-glucosyl units with a typical estimated mass of 10^6^ Da [14,16]. A part of the amylose molecules are linear containing a single long chain, whereas another part is slightly branched through α-(1,6) linkages. The number of chains in the branched amylose is only between approximately 5 and 20 [17], depending on the source of the starch, and the chains are shorter than in the linear fraction. Several chains are even as short as in amylopectin (i.e., DP < 100) [18]. Studies of the branching pattern of amylose molecules suggests a bimodal chain length distribution termed AM1 for relatively short chains of DP 100–700, and AM2 for longer chains of DP 700–40,000 [19] and that there is a species specific (genetic) component to variations in amylose fine structure [20]. The location of amylose in the starch granule is a subject of speculation [21], but X-ray scattering data suggest it is likely localized within the loosely organized amorphous regions of amylopectin blocklets [22], and that it may be more abundant near to the granule surface [23,24]. Early studies show that amylose, in preference to amylopectin, leaches from starches into water, suggesting a loose association with the amylopectin matrix [25]. Studies of *waxy* starches lacking amylose indicate that amylose imparts some degree of structural integrity to the granule, perhaps providing some degree of plasticization of the amylopectin component, since *waxy* starches are often prone to cracking and show reduced mechanical strength [26,27,28].

Besides the two major components, starch granules, notably those in plants with an altered starch biosynthesis (mutants), might also contain a third polyglucan component generally known as “intermediate material”, including material termed extra-long chain amylopectin [29]. The structure of this material is intermediate to that of amylose and amylopectin [30,31,32], but the exact structure has proved to be a challenge to understand. The structure of the intermediate material varies largely with the kind of mutation and with the species and variety of the plant wherein it is found.

### 2.3. Other Components

Starch granules also contain other minor components, such as lipids, minerals and proteins that may comprise up to a few percent by weight of the entire granule. Of these components, only phosphate is covalently linked to starch, and more precisely the amylopectin component. Lipids, especially lysophospholipids, are found in many cereals, in which a part forms inclusion complexes with amylose [33], and NMR studies suggest some may be associated with lipid-protein complexes [34]. Lipids are generally present in very small quantities in root and tuber starches. Instead, these latter starches are relatively abundant in phosphate groups covalently linked to the α-glucan chains of the amylopectin component. All starches and related polyglucans (e.g., glycogen) contain variable amounts of covalently-linked phosphate residues [35,36]. Many so-called B-crystalline starches tend to contain more covalently linked phosphate compared to A-crystalline starches. In particular, potato (*Solanum tuberosum* L.) starch is known for its relatively high content of phosphate groups, which is in the order of 300–900 ppm (and translates to a degree of substitution of roughly 0.1 to 0.3%). On the other hand cereal endosperm storage starches (A-crystalline) have the lowest starch phosphate contents, typically around <0.01% [37]. About two thirds of the phosphate is bound at the C6 position of the glucosyl units and between 20 to 30% at the C3 position [35]. A very small percentage of phosphate in starch is located at the C2 position [35,36]. Other elements than phosphorous are comparatively rare. These minerals are e.g., potassium, calcium, magnesium, and sodium [38,39]. In addition, starch granules contain a number of proteins bound within the water-insoluble amylopectin matrix, known as granule-associated proteins [40,41]. Most of these proteins are consistently found in granules, and at some stage involved in granule biosynthesis, remaining attached despite vigorous washing of starch granules with detergents and solvents. The manner in which granule-associated proteins become locked inside the starch granule is not clear and may vary depending on the protein. These proteins are mentioned in the sections below dealing with starch synthesis. In addition to the granule-associated proteins microtubules have been observed in some plant species to radiate from the central hilum forming proteinaceous channels, terminating at the surface of some starch granules and observed as holes or pores [42,43]. Actin-like and tubulin-like proteins are associated with these channels, including FtsZ, a conserved plastid division protein, and starch biosynthetic enzymes such as AGPase and SS isoforms [44]. The biological role of these channels is not known.

## 3. Architecture of Starch Granules

Starch granules exist in a variety of sizes and shapes depending on the plant species, and is reviewed in detail elsewhere [45,46,47,48]. Many root and tuber starches, such as potato and edible canna (*Canna edulis* L.) and starches from the bulbs of the orchid *Phajus grandifolius*, have large size-distributions of granules with sizes up to 100 μm or more [49,50], whereas in some cereals (e.g., rice) and pseudo-cereals (e.g., amaranth (*Amaranthus* sp.)) the size-distribution is in the order of 0.1-7 μm [51,52]. In other cereals, such as maize (*Zea mays* L.), the average size of the granules is intermediate around 10–20 μm [53,54]. Important members of the Pooideae (Poeae, Triticeae, and Aveneae) such as wheat (*Triticum aestivum* L.), oats (*Avena sativum* L.), rye (*Secale cereale* L.) and barley (*Hordeum vulgare* L.) are characterized by a bimodal size distribution of starch granules in the endosperm; large (also called A-granules) and small (B-) granules with approximate diameters of 12–35 and 2–10 μm, respectively [47,55,56], possibly controlled by the *B-GRANULE CONTENT 1* locus [57]. The shape of the granules is also highly variable depending on botanical origin, from round or polygonal (e.g., maize), oval (potato, peas (*Pisum sativum* L.)), disc shaped (A-granules in wheat and barley), to pyramidal (lesser yam (*Dioscoreaceae* species)) [45]. Some plant groups produce compound granules, e.g., rice (*Oryza sativa* L.) as well as members of Bambusoideae (bamboo) and Ehrharteae (veltgrass) [48]. There is evidence that in the grasses compound starch granules represent the ancestral starch granule morphology [58,59]. In *waxy* varieties the granules generally possess the same shapes as in the non-*waxy* counterparts of the species, whereas in high-amylose starches the shape of the granules tends to become irregular or elongated (e.g., high-amylose (*amylose-extender*) maize, amylose-only barley, or wrinkled (*rr*) peas) [53,60,61].

This reinforces the idea that the amylopectin component is the major contributor to the basic starch granule architecture in non-mutant (wild-type) plants. The mechanisms underpinning the various starch granule morphologies are not clearly understood. As our understanding of the processes governing granule initiation improves, it is becoming clear that elements of this machinery, as well as those of plastid division (e.g., FtsZ, see later sections) play a role in granule size determination [62]. In addition, different cell types and sizes may be important factors in granule size determination in addition to plastid tubule volume [63,64].

**Figure 1:**
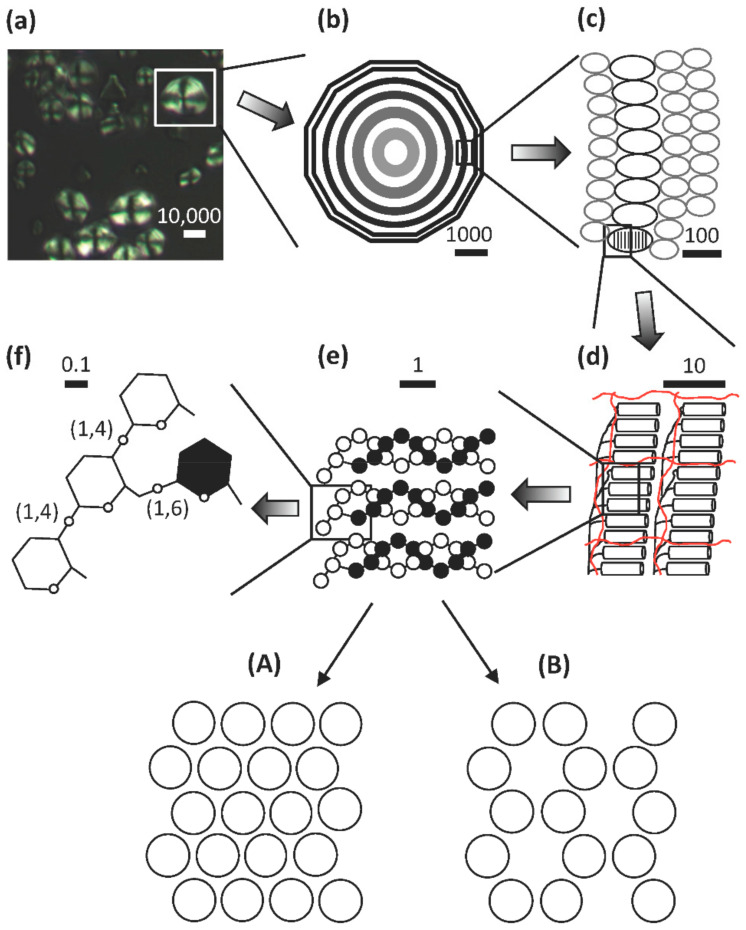
From granules to glucosyl units. (**a**) Maize starch granules observed under polarized light showing the characteristic “Maltese cross”. (**b**) A hypothetical granule with growth rings extending from the hilum. (**c**) Blocklets in semi-crystalline (black) and amorphous (grey) rings. (**d**) Crystalline and amorphous lamellae formed by double helices (cylinders) and branched segments of amylopectin (black lines), respectively. Amylose molecules (red lines) are interspersed among the amylopectin molecules. (**e**) Three double-helices of amylopectin. Each double-helix consists of two polyglucosyl chains, in which the glucosyl units are symbolized by black (A-chains, which are unsubstituted) and white circles (B-chains, which are substituted with other chains), respectively. The double helices form either A- or B-type allomorphic crystals (**A** and **B**, respectively, in which the circles symbolizes the double-helices seen from the edge). (**f**) Glucosyl units showing α-(1,4)- and α-(1,6)-linkages at the base of the double-helix. The bar scale (in nm) is only approximate to give an impression of the size dimensions. Reproduced from [14] with slight modifications.

Despite the large varieties in starch granule appearance, the internal architecture of the granules is remarkably conserved among plant species [65]. When viewed under the microscope, most granules (with maybe the smallest granules as exceptions) show a regular pattern of darker and lighter ring-like structures [50,66,67,68,69]. These are known as “growth rings”, granular rings, or shells and surround the so-called hilum or core of the starch granule (Figure 1), which generally is supposed to be the origin of starch granule biosynthesis (see section below on starch biosynthesis) [70]. This periodicity within granules is consistent across plant species and can also be analyzed using X-ray diffraction and electron microscopy [71,72]. The underlying reason for the existence of the granular rings and their constitution is uncertain. It was suggested in the older literature that the rings were the result of the diurnal cycle as they were found to be absent in wheat and barley plants grown in constant light conditions [73,74]. However, more recent investigations could not confirm these results as the rings remained in barley (both *waxy*- and wild-type barley) grown in constant light [69]. Also, in potato tuber the growth rings remained and it was suggested that the rings are formed due to a circadian rhythm instead [67].

The precise structural difference between the light and dark rings remain uncertain. It has been assumed that the rings consist of alternating amorphous (less dense) and crystalline (denser) regions, which would give rise to different refractive phenomena under the microscope. The crystalline, or semi-crystalline, rings presumably contain more ordered structures, to which the amylopectin component is the major contributor. Such ordered organization of the macromolecules in the granules is supported by the fact that starch granules are birefringent, i.e., they show a “Maltese cross” when observed under cross-polarized light (Figure 1), and that amorphous regions are lost faster under conditions of controlled digestion than ordered crystalline rings (see [10]). Atomic force microscopic investigations have shown bulb-like structures with diameters around 20–100 nm on the surface as well as in the interior of starch granules. These structures are known as blocklets and were isolated as discrete units from *waxy* maize starch [75]. The blocklets have been hypothesized to be a result of interactions of amylopectin super-helices [61], but their precise nature remains uncertain. Because of the dimensions, it has been assumed that the blocklets represent either single amylopectin molecules or smaller assemblies of the molecules, in which the amylopectin molecule is organized into stacks of alternating amorphous and crystalline lamellae with a repeat distance of about 9 to 10 nm, as judged by small-angle X-ray scattering (SAXS) (Figure 1). The 9 nm repeat distance of the lamellae corresponds to approximately 27–28 Glc units in double-helical conformation [76]. Blocklets containing stacks of these lamellae are thus building up the semi-crystalline rings. The amorphous rings were suggested by Tang et al. [77] also to contain the blocklets, however, in these rings the blocklets would be more "imperfect" in their architecture. Tang et al. [77] and Perez-Herrera et al. [75] further suggested that amylose is interspersed between and within the blocklets and extends over several blocklets in interaction with the amylopectin molecules. Amylose molecules are generally believed to be mostly found in the amorphous state in the granules (in the amorphous rings and/or amorphous lamellae), but they may penetrate the stacks of lamellae thereby imposing “imperfections” into the crystalline organization [22,78].

It is generally accepted that the crystalline lamellae are formed from self-assembled double-helices of short, external chains of amylopectin, whereas the majority of the α-(1,6) branch points in amylopectin are found in the amorphous lamellae (Figure 1). The double-helix has a diameter of 1.05 nm and the pitch of each of the parallel strands is 2.1 nm and consists of six glucosyl units [79]. The double-helices are organized into either a so-called A-allomorph or a B-allomorph crystalline pattern, which can be distinguished by wide-angle X-ray diffraction (WAXS, or XRD) [80,81,82]. The A-allomorph contains more closely packed double-helices and is typically found in e.g., cereal starches. The B-allomorph is less densely packed (essentially, one out of four double-helices is replaced by water molecules in the crystalline lattice) and is typically found in many tuber and root starches, which are more prone to turnover via amylolysis [83]. Some starch granules contain both types of allomorphs and gives a mixed pattern in WAXS. Such granules, known as “C-types”, are found in e.g., cassava (*Manihot esculenta* L.), banana (*Musa* sp.) fruits and many legume cotyledons [84,85,86]. The relative crystallinity in most starch granules is in the order of 20~45% [49,87,88,89]. *Waxy* starches are generally associated with somewhat higher values than the non-*waxy* counterparts, which suggest that the amylose component does not contribute to the crystallites to any notable degree. However, it should be noted that the external chain segments in amylopectin generally constitute about 60% of the macromolecule. Cook and Gidley [90] found that the amount of double-helices is larger than the degree of crystallinity and, as the degree of crystallinity hardly exceeds 45% even in *waxy* starches, this suggests that several of the external segments are not necessarily organized into crystallites, albeit forming double-helices.

## 4. The Amylopectin Backbone Model

The unit chains of amylopectin are of two major types, namely short and long chains. Short chains are defined as chains with DP 6 to 36 (but the upper limit depends on the source of amylopectin) [89]. Long chains have DP ≥ 36 and the unit chain distribution can be obtained for analysis by enzymatic debranching of the macromolecule and subsequent separation of the debranched products by size-exclusion chromatography (SEC) [7,91] or high-performance anion-exchange chromatography (HPAEC) [92,93], or by fluorophore-assisted capillary electrophoresis (FACE) [94]. The molar distribution of short to long chains (S:L) is in the order of 6–19 in most starches: generally lower in B-crystalline starches than in A-crystalline types [89]. The chains are also traditionally divided into A-, B- and C-chains [95]: A-chains are not substituted by other chains, whereas B-chains are substituted by other chains (A- or/and B-chains). In addition, each macromolecule consists of one C-chain, which carries the sole reducing-end group but otherwise is similar to the B-chains. Furthermore, chain segments found outermost in the macromolecule, i.e., between the outermost branch points and the non-reducing ends, are considered as external chains, whereas chain segments between branches are internal chains (including the chain segment between the innermost branch point and the reducing end) [96]. A total internal chain segment equals the whole chain except the external segment (i.e., it contains all internal segments and all branch points) [97]. By definition, therefore, all A-chains are completely external, whereas all B-chains (and the sole C-chain) contain one external segment and a total internal chain segment (which is divided into one or more internal chain segments).

**Figure 2:**
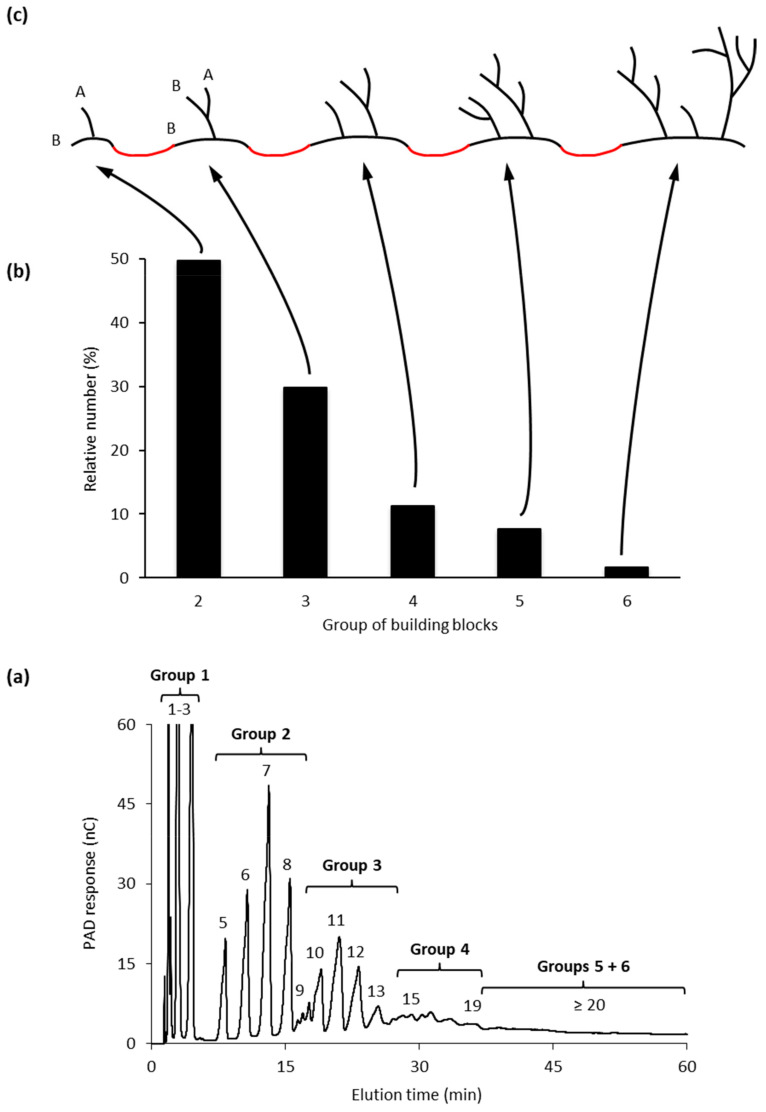
Composition of building block units in amylopectin. (**a**) The size distribution of building blocks from mung bean cotyledon amylopectin was determined by HPAEC-PAD and obtained by α-amylolysis and successive β-amylolysis. Groups of building blocks and DP of the peaks are indicated. Group 1 contains Glc, maltose and maltotriose, Groups 2–6 are branched dextrins, of which individual peaks of Groups 5 and 6 are not resolved by HPAEC. (**b**) The relative number of branched building blocks of the respective groups. The PAD response was calibrated to quantitative amounts for each DP. (**c**) The principal structures of the branched building blocks with A- or B-chains indicated for groups 2 and 3. Group 5 contains building blocks with 5–7 chains and group 6 has ≥8 chains. The multitude of possible structures of building blocks increases rapidly with the number of chains. Red lines between the building blocks symbolize the inter-block segments.

The structure of amylopectin is ultimately determined by the organization of its unit chains. This means that determining the organization of long and short chains, A- and B-chains, the C-chain, as well as the external and internal segments, is of fundamental importance to understanding the macromolecular structure. The most straightforward way to unravel the organization is to isolate the branched structural units of the macromolecule. For this purpose the endo-acting α-amylase of *Bacillus amyloliquefaciens* has been used [97,98,99]. *B. amyloliquefaciens* α-amylase preferentially attacks longer chain segments between branch points, but internal segments with three or less residues are resistant to attack (and maybe also those with four residues) [100]. The enzyme attacks, however, also external chain segments and reduces their lengths (ECL) to an average of approximately 2 residues [101]. The resulting branched limit dextrins contain branch points in close proximity to each other, i.e., their internal chain lengths (ICL) is only ≤3 residues. These limit dextrins represent the branched structural units of amylopectin and have been named building blocks [102] (see Figure 2).

The size-distribution of the building blocks have been analyzed by SEC and HPAEC and it has been shown that the size-distribution is remarkably similar regardless the botanical source of amylopectin [103]. Building blocks can be isolated, structurally characterized, and divided into different groups depending on the number of chains that they contain (Figure 2a, and [103]).

Group 2 building blocks consists of only two chains (and thus a single branch point), i.e., they have an A-chain bound to a B-chain (though in this case more correctly defined as a C-chain; Figure 2c) and have a DP range of between 5 and 9. Group 3 building blocks consist of three chains (and thus two branches) and have a DP range of 10 to 14, whereas building blocks in Group 4 have four chains (DP 15 to 19) (Figure 2). Building blocks of Group 5 are isolated as mixtures of dextrins with DP 20 to 35 and consist on average of six chains, whereas Group 6 (DP > 35) contains 9–12 chains [103].

The most common group of building blocks found in all amylopectins is Group 2 and comprises approximately 50% by number of all the blocks (Figure 2b) and about 25% of all branch points in amylopectin are found in this group. The second most abundant group is Group 3, to which about 30% of the blocks belong. This group represents another approximately 25% of the branch points of amylopectin. Building blocks of Group 3 have either of two possible principal structural configurations, known as the Haworth (one A- and two B-chains, [104]) and the Staudinger configurations (two A- and one B-chain, [105]), the former of which is shown in Figure 2c. Most probably there is a mixture of these configurations in amylopectin, but the mixture might be different in different starches. Group 4 comprises approximately 10% of the building blocks by number, and the possible combinations of the chains in the dextrins increases rapidly with the number of chains. Group 5 represent 5 to 8% of the building blocks and the number of individual dextrins is so large so that individual peaks are not resolved by HPAEC, but they can be quantitatively measured by SEC. Finally, group 6 is represented by only a few percent of all the blocks and approximately 16% of all branch points are included within this group.

The individual α-glucan chains in the building blocks are very short. A peak at DP 2 or 3 (mostly A-chains) is obtained by HPAEC and another peak at DP 5, 6 or 7 (B-chains) for most starches. The lengths of the longest chains increase slightly with the number of chains included in the groups. Group 6 contains apparently another, longer type of chains with a peak at around DP 8. It was suggested that this could be due to a combination of two building blocks being remnants of an “incomplete debranching” during amylopectin synthesis, which would result in the combination of two smaller building blocks (discussed later) [103]. The ICL in building blocks is short: on average between 1.2 and 2.2 glucosyl units. The total internal chain length (TICL) is between, 4.1 and 5.8, and increases with the size of the blocks [103].

The building blocks are interconnected through inter-block segments (IB-S, shown in red in Figure 2c). The lengths of these segments have been estimated by analyzing the number and structure of building blocks contained within larger α-dextrins that consist of two or more building blocks. When the *B. amyloliquefaciens* α-amylase attacks between building blocks, very small, linear saccharides (Glc, maltose and maltotriose) are obtained as “Group 1” (Figure 2a), and the amount of these saccharides is a measure of the inter-block chain length (IB-CL) [9,106]. IB-CL varies mostly between 5 and 8 Glc residues [107], but a smaller number of IB-Ss appear to be longer, maybe up to 15 or 20 residues [9,106,108,109]. Thus, as many of the branch points (25%) are contained within Group 2 building blocks, these single branch points are separated by IB-CL of 5 to 8 residues, or more. Up to 25% of the branches are found as pairs (with ICL 1–3 in Group 3) which are also separated by IB-CL 5 to 8. The rest of the branches are found as slightly larger groups with ICL 1 to 3 and these groups are also separated by the IB-Ss. Only a minority of the branch points (16%) is found “clustered” by ICL of 1 to 3 within Group 6.

**Figure 3:**
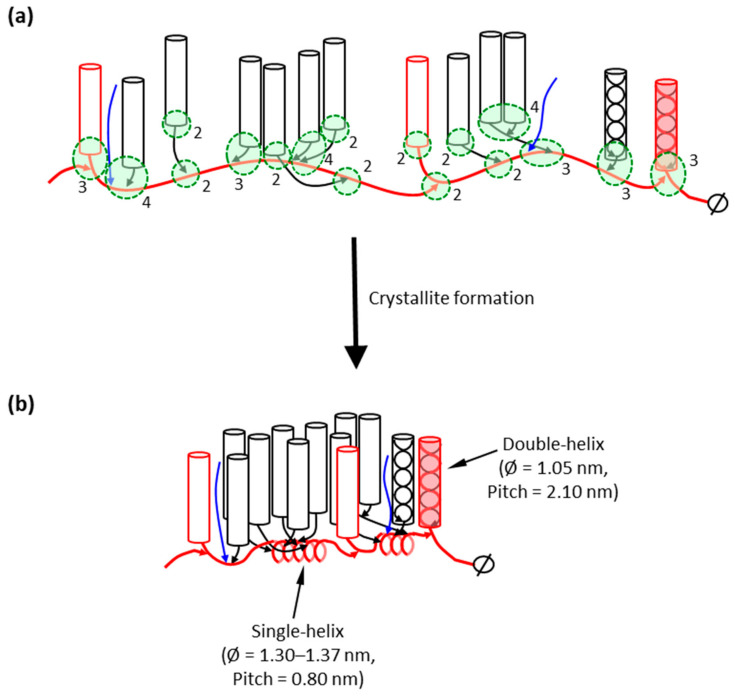
Amylopectin backbone structure. (**a**) The principle structure showing the backbone (red) with characteristic inter-block segments between the branched building blocks (encircled in green). Numbers indicate the groups of building blocks of which group 2 has two chains, group 3 has three chains and group 4 has four chains (the minor groups 5 and 6 are not depicted in the figure). The short chains form double-helices (cylinders). Also external segments of the long chains forming the backbone participate in double-helices (red cylinders). Some chains do not form double helices (blue chains) and presumably introduce distortions among the double-helices, which affects the formation of crystallites. (**b**) The backbone is flexible and may form singe-helical segments, which contract the macromolecule and brings individual double-helices together enabling them to crystallize. To form larger crystallites with either the characteristic A- or B-allomorphs, double-helices from several individual amylopectin molecules have to come in proximity, however. The dimensions of the single- and double-helices are based on data given by Zobel et al. [112] and Imberty et al. [79], respectively.

The question arises as to where in the amylopectin macromolecule the IB-Ss are found. Very small groups of building blocks (α-dextrins) that have been isolated do not contain longer chains, but somewhat larger groups, with three or more building blocks, tend to contain longer chains [107,110]. This strongly suggests that the major part of the building blocks is interconnected by the long chains of amylopectin. The most probable structural model of amylopectin is therefore that the major part of the building blocks is outspread along these long chains of amylopectin (Figure 3a). These long B-chains are presumably interconnected together through α-(1,6)-linkages forming a longer backbone in the macromolecule. The constitution of building blocks from different groups along the backbone is apparently completely random [111]. It was suggested that shorter B-chains (DP < 36) are forming short branches that interconnect “external” building blocks to the backbone (Figure 3a). Some amylopectins, mostly A-crystalline storage starches mainly from cereal endosperms (with high S:L), might have more of these branches and also contain some shorter B-chains within the backbone, than amylopectin in B-crystalline starches (low S:L) [107].

The backbone of amylopectin is envisioned to be a flexible structure. The longer the inter-block segments, the more flexibility they introduce into the backbone, which has an important impact on starch properties and functionality [14,113,114]. It was recently demonstrated that the internal chains of amylopectin have the capability of binding iodine (I_3_^−^) [115,116]. This showed that the segments of the backbone can form helical inclusion complexes with iodine and, possibly, helical segments also exist in native starch even without the complexing agent. These segments are probably the inter-block segments, the length of which theoretically could form single-helices with approximately six residues per turn (Figure 3b). This would allow double-helices along the backbone to come into close proximity and promote interaction between the double-helices, e.g., the formation of crystallites.

Besides the two major groups of short and long chains, amylopectin from some plants, e.g., *indica* rice varieties, wheat, potato and cassava, also contains “superlong” (or extra long) chains [117,118,119,120]. The superlong chains contain hundreds or up to thousands of glucosyl units and have thereby similar lengths as the amylose component. The superlong chains appear to carry a few long branches, resembling branched amylose [117]. The position of superlong chains within the amylopectin macromolecule is not known, but one could believe that they are associated with the backbone, albeit without carrying double-helices. The amount of superlong chains varies greatly from a few percent by weight in e.g., wheat, potato and cassava [118,119,120] to more than 10% in *indica* varieties of rice [121]. Superlong chains are not found in *waxy* starches, however, and the enzyme responsible for the synthesis of these chains is granule-bound starch synthase I (GBSS I) [122], i.e., the same enzyme that synthesizes amylose and which is inactive or absent in *waxy* plants.

## 5. The Backbone Model in Relation to the Granular Structure

**Figure 4:**
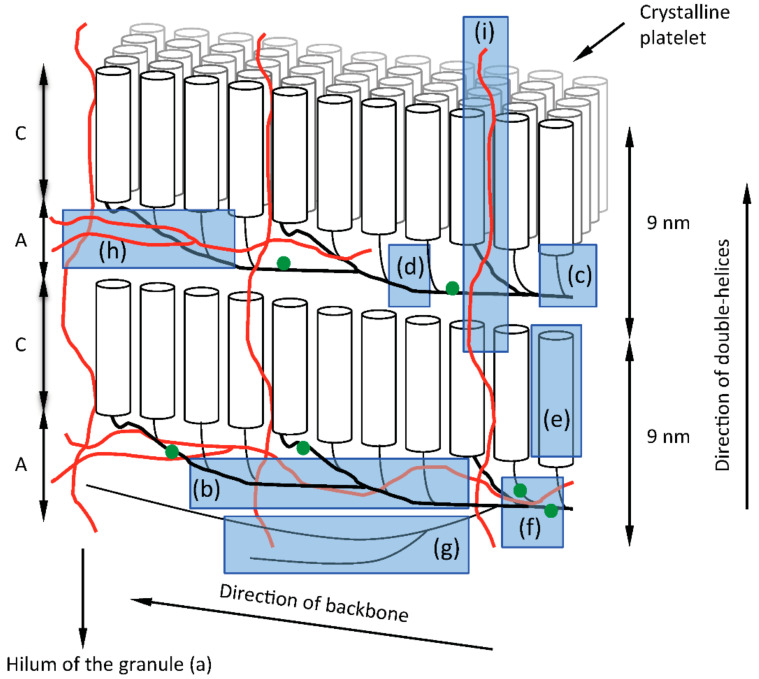
A cartoon of the building block backbone model of amylopectin with its structural elements in the starch granule along with the possible involvement of amylose. Stacks of amorphous (A) and crystalline lamellae (C) are indicated with a repeat distance of approximately 9 nm. Layers of amylopectin molecules are laid upon each other during the biosynthesis and crystalline platelets are formed by the double-helices (cylinders) as indicated in the 3D-view. The non-reducing ends of the double-helices are directed toward the granule surface and the hilum of the granule (**a**) is found in the other direction. Amylose (red lines) is suggested to be found interspersed with the backbone of amylopectin (bold black lines) in the amorphous layers, which increases the stability of the starch granules. Some amylose molecules are also crossing the layers, thereby providing additional stability to the layered structure. The structural elements are highlighted in blue as (b) the backbone, (**c**) building blocks, (**d**) inter-block segments, (**e**) external segments (forming double-helices), (**f**) phosphate groups (green dots), (**g**) super-long chains, (**h**) branched amylose, and (**i**) linear amylose. See also Table 1 which refers to the same structural elements in relation the respective enzymatic reactions responsible for their synthesis. Note that the actual positions of phosphate groups remain uncertain.

As any model, the backbone model has to be accommodated into the structure of starch granules as far as it is accepted to date. Based on the model, the amylopectin macromolecule builds up the semi-crystalline layers by being laid down layer by layer during starch biosynthesis, so that each layer of amorphous and crystalline lamella is formed simultaneously during the formation of the amylopectin molecule (Figure 4). The direction of growth of the entire macromolecule is tangential following the direction of the backbone, which is included into the amorphous lamellae, whereas the direction of the double-helices, formed by the short chains of amylopectin, is envisioned in a more or less perpendicular direction to the backbone [10].

The external segments of the long backbone chains might, however, participate in the crystalline lamella as well, as it has been strongly indicated that the lengths of external segments of long B-chains closely corresponds to the average ECL of amylopectin [89], and thus probably form double-helices just like the short chains do. Even if in this model the backbone of amylopectin is directed tangentially in the granules, the vast majority of the chains (the double-helices) are found in the radial direction and are responsible for the characteristic ”Maltese cross” observed when starch granules are viewed under cross-polarized light (as shown by the fact that the amorphous parts of the granule can be removed without the loss of the cross [60,123]. It is also noted that the biosynthesis of the layers of the starch granule is by apposition with this model, in agreement with experimental results [124], as the majority (about 90%) of the chains of amylopectin are synthesized in the radial direction.

It is noted that with the backbone model the double-helices are directed radially in the granule in the same fashion as with the traditional cluster model, however the direction of the backbone (and thus the macromolecule) is tangential as opposed to the cluster model, in which the entire macromolecule is directed radially [125]. The direction for the latter model was primarily based on the birefringent pattern (the “Maltese cross”) and the fact that the synthesis of the granule is by apposition [5]. Hizukuri [7], who found a polymodal size-distribution of the unit chains in amylopectin and a periodicity in chain length of 27 to 28 glucosyl units using size-exclusion chromatography, suggested that B2-chains penetrate through two clusters and B3-chains through three clusters, etc., and that each cluster would be a part of the repeating crystalline and amorphous lamellae, i.e., each cluster corresponds to the repeat distance of 9 nm, which in turn corresponds to the found periodicity in chain length—if it is assumed that the glucosidic chains extend both through the crystalline and the amorphous lamellae in double-helical conformation. However, no experimental results so far have suggested that this is the case. Rather, the sparse information on the structure of the amorphous lamellae indicates that chains in the amorphous lamellae might be found in all directions and possibly obtain partial single-helical conformations [115,116,126]. It is also important to note that Hanashiro et al., who used ion-exchange chromatography, did not find a periodicity in chain length among the long chains of amylopectin (DP > 36) (albeit they found a much shorter periodicity of DP 12 among the short unit chains) [92]. Indeed, Palmer et al. [127] highlighted that if the unit chain distribution is drawn on a numerical (molar) basis rather than weight basis, the long chains are not found as a clearly separate group of chains, but as a “shoulder” to the short, major group of chains. It therefore appears that the method used for analysis (SEC and type of column, HPAEC, FACE) and the way of presentation of the results, impacts on the impression of the existence of diverse chain categories. The nomenclature of the α-glucan chains used for the backbone model, B2- and B3-chains (as well as B1- and A-chains) are adapted from the previous literature in order to distinguish chain-length categories, but their structural function is different from that in the cluster model. For most amylopectins, B2-chains constitute the major part of the long chains, whereas B3-chains only form a tail to this group, showing that there exists a minor part of long chains in decreasing number with increasing length and with no periodicity in length [89]. Thus, a major part of the chains in the backbone are classified as B2-chains but occasionally longer chains are also found there.

It has been known for a long time that the average chain length (CL) of amylopectin correlates with the crystalline allomorphs of starch granules. Thus, shorter CL results in the A-allomorph and longer CL gives the B-allomorph, whereas intermediate CL tend to give the C-allomorph pattern [7]. However, it should be noted that the double-helices comprise only the external segments of the chains and the proportion of both the external and internal chains tends to remain approximately the same in most amylopectins as shown by the Φ, β- amylolysis (or β-amylolysis) limit values, which are around 50 to 60% [89]. Thus, a longer CL also implies longer ECL as well as longer ICL values. The chain length requirements of the two internal chain segments (m and n, of which m is the internal segment of the main chain and n is the internal segment of the side chain connecting to another double-helix) connecting two double-helices arranged in the crystalline lattice were investigated and it was found that only very restricted chain length combinations of the two segments gave rise to either the A- or B-allomorph [126]. Only the combination of ICL of *m* = 1 and *n* = 3 or *m* = 4 and *n* = 6 could give the A-allomorph structure, whereas the latter together with *m* = 6 and *n* = 4 results in the B-allomorph. In addition, *m* = *n* = 7 could give rise to two parallel standing double-helices. Any other combination of m and n did not give parallel double-helices. This very restricted combination of internal chain lengths would result in restricted chain length distributions of the short internal chains of amylopectin. However, the internal chain length distributions in isolated limit dextrins show that all chain lengths within the DP-interval 3 to 7 are quite common and therefore the results obtained by O’Sullivan and Pérez [126] appears to only represent minor types of double-helix combinations. Vamadevan et al. [113] found a correlation between the inter-block chain length (IB-CL) and the onset gelatinization temperature of starch from different plants. Shorter IB-CL, found generally in A-crystalline starches, tend to correlate with low onset temperatures, whereas long IB-CL, typical for B-crystalline starches, tend to increase the temperature and thus stabilize crystalline structures. It should be noted, however, that exceptions to this generalization exist, as Vamadevan et al. [113] also found that potato and edible canna starch have comparatively low onset gelatinization temperatures despite possessing long IB-CL. Nevertheless, this implies that the internal chains of amylopectin, in particular the inter-block segments, contribute to the type and stability of the crystals: longer segments introduce more flexibility into the structure and thereby stability [128]. The results suggest therefore that the internal structure of amylopectin is of importance for the type of crystal allomorph in the granule. However, in this context it is interesting to note that black pepper (*Piper nigrum* L.) amylopectin appears to be an exception as it was found to have comparatively short ECL and possesses A-crystalline granules despite unusually long average ICL [129]. Unfortunately, the actual IB-CL of black pepper amylopectin was not analyzed.

Crystalline lamellae have been isolated from acid-treated *waxy* maize starch granules as discrete platelets [130] with dimensions suggesting that they include hundreds of double-helices [21]. Such large assemblies suggest that each platelet might be a composite of double-helices from several individual amylopectin macromolecules. Alternatively, the double-helices in the platelets could be arranged into the large crystalline arrays if the backbone of single macromolecules folds so that distant helices along the backbone come in close proximity and co-crystalize. Oostergetel and van Bruggen [72], who analyzed the structure of potato starch granules using electron optical tomography and cryo-electron diffraction techniques, found that amylopectin forms super-helical structures containing the stacks of alternating crystalline and amorphous lamellae. They depicted the superhelices as formed collectively by several amylopectin molecules (having the cluster structure). Later, Waigh et al., using SAXS with microfocus scattering, depicted the super-helix as formed by a single macromolecule with “pie-shaped” crystalline lamellar motifs [71] possibly corresponding to the later isolated platelets [130]. If the super-helix indeed is built up from a single macromolecule, a backbone structure of amylopectin seems likely. Interestingly, such super-helices might correspond to the blocklets in the granules. Recently a blocklet was modeled based on the dimensions of the isolated platelets and following phyllotaxic rules [131], with the results of a cigar-like structure having similar dimensions as the isolated blocklets from *waxy* maize starch [75]. It was calculated that the size of the modelled blocklet corresponds to a single amylopectin molecule with a molecular weight in the range of 10^9^ Da [131].

To summarize, the structural elements of the granule and the *building back backbone* model of amylopectin are highlighted in Figure 4: (a) The hilum of the granules represents the origin of biosynthesis and is surrounded by the regular lamellar organization of amylopectin molecules. (b) The backbone consists of inter-linked long B-chains found in the amorphous lamellae of the granules. (c) Building blocks are outspread along the backbone and represent single or tightly branched units with mostly two or a few more branch points. (d) Flexible inter-block segments between the building blocks have 5 to 8 or more glucosyl units and have the capability to form single-helical conformation. (e) Short chains extend from the building blocks into the crystalline lamellae where they form double-helices. (f) Phosphate groups are esterified at C6 and C3 positions on the glucosyl units presumably mostly along the long chains of the backbone to varying degrees in different types of starches. (g) Superlong chains might be attached to the backbone. (h) Branched amylose is presumably mostly interspersed among the backbone of amylopectin. (i) Linear amylose might penetrate the lamellar stacks and impose structural defects into the crystallites. (Note that the positions of branched and linear amyloses are based on the recent proposal by Vamadevan and Bertoft [132], however no direct evidence for this exists.) Each of these structural elements are synthesized by one or more specific enzymes or enzyme complexes as indicated in Table 1 and further discussed below.

## 6. Biosynthesis of the Starch Granule

The elaborate architecture of the starch granule is matched by the complexity of its biosynthesis, which involves more than fifteen enzymes, many of which are regulated by various post-translational control mechanisms [132,133]. An unusual feature of this biochemical pathway is the fact that soluble precursors, ADP-glucose (ADP-Glc) and malto-oligosaccharides (MOS) are utilized and fashioned into a water-insoluble macromolecular structure whose three-dimensional features and solubility are continuously altering from initiation to completion of the granule structure. Attempts at understanding this process through modelling have been reviewed recently [134,135]. Following formation of ADP-Glc, the components of starch, amylose and amylopectin are synthesized inside plastids via the actions of three major groups of enzyme activity, each comprising of multiple tissue- and developmental-specific isoforms; these are starch synthases (SS), starch branching enzymes (SBE), and starch debranching enzymes (DBE).

**Figure 5:**
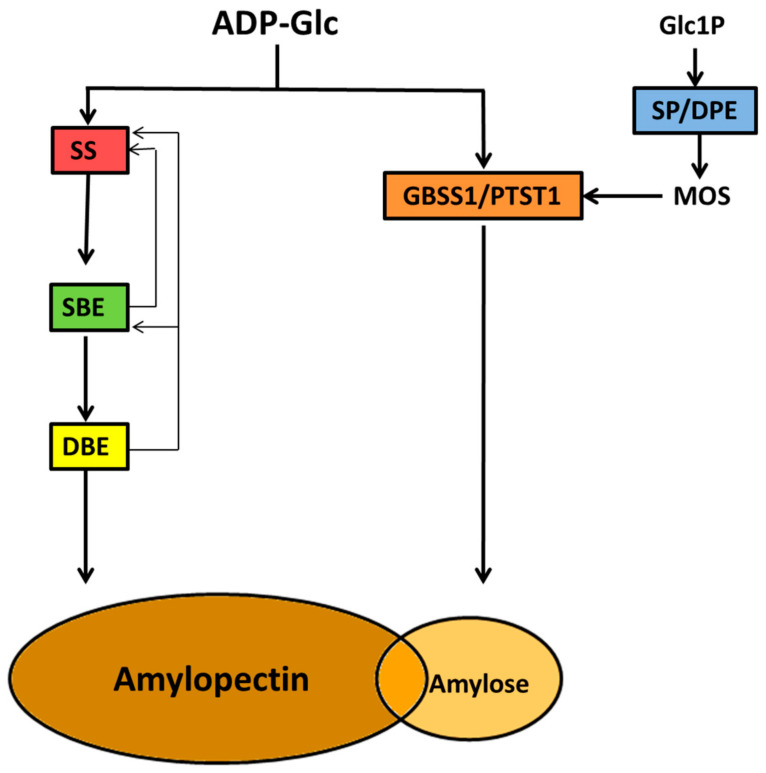
Generalized schematic of the pathway of starch biosynthesis following granule initiation and hilum formation showing the major enzyme classes (in boxes) responsible for granule structure. ADP-Glc is the immediate soluble precursor for starch synthesis and is utilized by soluble SS isoforms which create extended α-(1,4) linear glucan chains in amylopectin by addition of Glc from ADP-Glc. ADP-Glc is also utilized by GBSSI for amylose synthesis, a process requiring MOS primers. MOS may arise from the activities of SP and DPE as well as DBEs during amylopectin trimming. Amylopectin arises through the combined actions of multiple isoforms of SS, SBEs (introduction of α-(1,6) branch linkages) and DBEs (removal of selected α-(1,6) branch linkages), with each enzyme class potentially using substrates and products of others. Typically starches contain approximately 75% amylopectin, although some of this material is made up of relatively long and sparsely branched α-(1,4)-linked chains (super-long chains) intermediate between amylose and amylopectin depicted as the area between the overlapping regions of amylose and amylopectin in the figure.

A simplified starch biosynthesis scheme is presented in Figure 5 and outlines the major enzyme classes involved in amylopectin and amylose synthesis following the initiation steps which produce the hilum. In addition, other enzyme activities are also involved, including a number of recently characterized non-catalytic proteins. The following sections discuss these enzymes and outline, where possible, the relationship between the coordinated activities of enzymes in the biochemical pathway with the final product of the starch granule based on the *building block-backbone* model of the amylopectin structure.

## 7. Commitment of Carbon for Starch Biosynthesis

The first committed biochemical step of starch biosynthesis is the formation of the immediate soluble precursor, ADP-Glc by ADP-Glc pyrophosphorylase (AGPase) from Glc1P and ATP [136]. The importance of this reaction lies in the fact that it a controlling point in the partitioning of carbon from the hexose-phosphate pool of general metabolism into starch. Although ADP-Glc levels have been shown to influence amylose: amylopectin contents [137], the AGPase reaction plays no direct role in determining starch structure. Details of the regulation of ADP-Glc synthesis by AGPase are therefore covered elsewhere [48,132], since starch granule formation *per se* begins with the formation of linear *α*-glucans from ADP-Glc by the various starch synthase isoforms (SS).

## 8. Starch Granule Initiation

Our understanding of how starch granules are initiated is currently an emerging, and active field of research. Unlike eukaryotic glycogen synthesis, which requires the proteinaceous primer glycogenin to initiate the formation of particles [138,139,140,141], starch granule initiation appears to be the result of the combined activity of a network of catalytic and non-catalytic proteins. The starch granule initiation pathway is outlined below, but a more detailed account of this subject can be found in recent reviews [142,143].

Starch granule initiation requires either de novo synthesis of MOS from soluble sugars (e.g., in cells producing storage starches), or building of semi-crystalline competent amylopectin from extant structures (e.g., transient starch in leaf cells), where in the latter case a residue of the granule often remains after the dark period [144]. The extent to which a starch granule is fully degraded at night, or barely at all in the case of storage starches, is subject to many factors including environment, tissue/cell type and age, and is suggestive of an as yet uncharacterized cellular signaling pathway [145]. Non-catalytic proteins associated with the mature starch granule have been implicated in the regulation of nocturnal degradation rate. Studies with Arabidopsis (*Arabidopsis thaliana* L.) by Feike et al. discovered two related non-catalytic starch binding proteins, early starvation 1 (ESV1 and like early starvation 1 (LESV1)) [146]. ESV1 and LESV1 are stromal proteins found bound to starch granules via tryptophan-rich carbohydrate binding regions, and common to all plants and green algae [146]. ESV1 and LESV1 may regulate starch degradation via α-glucan modification at the granule surface.

### 8.1. Proteins Associated with Granule Initiation

It is generally accepted that the growing starch granule emanates from a central core known as the hilum (Table 1). The precise structure of the hilum is unknown, but X-ray data suggest a relatively disordered α-glucan structure [81,147,148]. Initiation of the hilum, and the subsequent formation of normal granules requires a single SS isoform (SSIV) [149]. Studies in Arabidopsis suggest that SSIII may also play a role in initiation as it appears there is some overlap in its action with SSIV in this regard [150,151]. SSIV plays a unique role in initiation by interacting with a class of non-catalytic proteins which were previously identified as potential regulatory scaffold proteins [152]. Seung et al. [153] showed that in Arabidopsis SSIV interacts with a protein termed PROTEIN TARGETING TO STARCH 2 (PTST2), part of a family of PTST proteins characterized by the presence of coiled-coil protein interaction domains and a family 48 carbohydrate binding (CBM48) domain at the C-terminus which facilitates binding to α-glucans [154]. The ability of SSIV to dimerize appears to be important for catalytic activity and the ability to form interactions with other proteins [155]. In the model put forward by Seung et al. [153] PTST2 recognizes MOS of a specific three-dimensional (helical) shape via its CBM48. The PTST2/MOS complex then interacts with a SSIV dimer allowing SSIV to elongate the α-glucan, possibly releasing PTST2 and allowing it to bind to other helical MOS structures for further interaction with SSIV. In this proposed scheme aggregated, semi-soluble α-glucan structures are formed which can evade the degradative activities of α- and β-amylases.

SSIV requires pre-existing α-glucan chains for elongation and is particularly active with maltotriose [150], which begs the question of the source of α-glucan primer for starch granule initiation. SSIII has been shown to be capable of unprimed α-glucan formation in the presence of ADP-Glc [150], and starch phosphorylase (SP, also termed Pho1) is capable of producing and extending MOS in the absence of α-glucan primer using Glc1P [156]. The potential priming reactions by SSIII and SP have been cited as important components of the granule initiation pathway [157,158,159]. Studies with rice endosperm show that SP forms a protein complex with disproportionating enzyme (DPE) [160] and the SP/DPE complex may provide MOS substrates for other components of the granule initiation machinery [142]. DPE may well play a role in both the granule initiation process as well as subsequent steps in starch synthesis as it is also involved in modification of MOS chain length [160,161].

In addition to the PTST family, other non-catalytic proteins are implicated in the starch granule initiation pathway, and appear to play important roles in protein scaffolding and sub-organellar localization. A novel chloroplast protein discovered in Arabidopsis known as PROTEIN INVOLVED IN STARCH INITIATION1 (PII1, At4g32190) forms a protein complex with SSIV, and may be necessary for SSIV catalytic activity [162]. PII1 is also referred to as MYOSIN-RESEMBLING CHLOROPLAST PROTEIN (MRC), which was identified as one of two proteins interacting with PTST2 by Seung et al. [163]. This recent study by Seung et al. showed that PTST2 interacts with both PII1 (MRC) and thylakoid-associated MAR-BINDING FILAMENT-LIKE PROTEIN (MFP1), both coiled-coil containing proteins. Much of the granule initiation machinery has been deduced from studies in Arabidopsis, and it is not clear if similar non-catalytic proteins are required for localizing storage starch granule formation within non-photosynthetic plastids such as amyloplasts. Most recently, another component of the granule initiation machinery has been discovered in Arabidopsis. SSV appears to regulate the number of starch granules formed inside plastids acting through an α-glucan binding domain and interacting with PII1/MRC. Interestingly, SSV lacks glycosyltransferase activity and is closely related to SSIV; *ssv* mutants show reduced numbers of starch granules per chloroplast but larger granules, indicating that other components of the granule initiation machinery can compensate for loss of SSV to some degree [164].

### 8.2. Contribution of the Initiation Machinery to Granule Structure

As discussed above, the hilum appears to be structurally disorganized, but is sufficiently resilient to degradation to act as a glucan scaffold for continued granule growth by the main classes of enzymes involved in amylopectin biosynthesis. It is important to note that the combined activities of the granule initiation machinery as outlined above produce linear unbranched MOS and the beginnings of the hilum from which the nascent starch granule grows. Soluble MOS can be formed inside plastids from a number of enzyme activities, including those associated with the granule initiation machinery, such as SS isoforms and starch phosphorylase which form linear, unbranched MOS. It is therefore speculated that the association of SSIV with PTST2, PII1/MRC and SSV produce a micro-environment conducive to formation of semi-crystalline α-glucans. Linear MOS can self-assemble to form helical coils, as such conformations arise spontaneously when linear MOS reach a critical DP [165,166]. Such coiled MOS structures may be the first water-insoluble structures formed which facilitate starch granule initiation, and therefore less prone to degradation by α- and β-amylases, particularly if physically shielded by the protein complexes associated with the initiation machinery [142]. At some point during granule formation these linear MOS structures must be branched by starch branching enzymes (SBE). It is not known specifically when SBEs act in the formation or extension of the hilum structure. Studies with barley endosperm at very early stages of endosperm development (0 to 1 days after anthesis) show that SBEIIa is the only branching enzyme expressed, suggesting a role for this enzyme in granule initiation [158]. In addition to its possible priming role, SP may work in conjunction with SSs and SBEs to produce an initiation point for continued growth of the hilum from the structures arising from the SSIV/PTST2 interaction [167] to begin the formation of the amylopectin backbone and subsequent building blocks.

## 9. Amylose Synthesis

### 9.1. Granule Bound Starch Synthase

A single enzyme, known as granule bound starch synthase (GBSS) (Table 1), is responsible for amylose biosynthesis in plants and green algae and is encoded by the *Waxy* locus as loss of catalytic activity results in an amylose-free, or *waxy* starch [168,169]. The synthesis of amylose and amylopectin is not entirely coeval, since amylose deposition occurs inside a pre-existing starch granule matrix, and requires the water-insoluble amylopectin scaffold to target GBSS to the granule. Indeed, it is thought that the processive mode of action peculiar to GBSSs is dependent on the presence of amylopectin [170]. Consequently, in many storage tissues the amylose content of starch gradually increases during development of the tissue [171,172]. GBSS is structurally related to other SS isoforms (for reviews see [41,173]) and is the most abundant protein found inside starch granules among the other granule-associated proteins [41,174]. Two tissue-specific isoforms of GBSS exist which are encoded by separate genes, GBSSI expressed in storage (non-photosynthetic) tissues, and GBSSII found in chloroplasts involved in transient starch synthesis in photosynthetic tissues [175], and there is evidence that slight species-specific differences in the products of GBSSs exists [176]. GBSS acts in a processive manner, adding Glc from ADP-Glc onto the non-reducing end of an α-(1,4)-linked glucan chain [170]. GBSS catalytic activity is stimulated by the presence of MOS [177,178] and there is evidence that the enzyme is regulated by protein phosphorylation [179,180,181] and appears to form oligomers in the presence of its soluble substrate ADP-Glc [182]. The precise role protein phosphorylation and oligomerization play in regulating GBSS is not known. In addition to the catalytic activity of GBSS, the formation of amylose requires a member of the PTST family (PTST1) in order to target GBSS to the starch granule [183]. PTST1 has no catalytic activity, and was formerly identified as scaffolding protein At5g39790 in Arabidopsis [152]. PTST1 acts by interacting with GBSS through conserved coiled-coil domains and trafficking the complex to the starch granule via a CBM48 [153,183]. Loss of PTST1 in Arabidopsis results in loss of GBSSI from the starch granules and a *waxy* phenotype [183], and its conservation throughout the plant Kingdoms argues for a critical role in amylose biosynthesis. However, studies in barley and rice endosperm suggest the impact of loss of PTST1 is greatest in chloroplasts, since effects on amylose synthesis in endosperm amyloplasts were highly variable [184,185].

A proportion of amylose contains α-(1,6)-branch linkages, but these branch points are sparsely distributed and not clustered as in amylopectin [18]; the biological function of this process is not known. The enzymes responsible for branching amylose are not known, however, in vitro studies with SBEs indicate SBEI has a preference for amylose, suggesting this isoform may be involved in vivo [186,187].

### 9.2. Contribution of GBSS/PTST to the Building Block-Backbone Model

The distribution of amylose in the starch granule is uneven, being found more conspicuously at the granule periphery and the equatorial crease/groove of growing granules [188]. Since GBSS synthesizes amylose and related material (e.g., super-long chains [28]) within an extant amylopectin matrix, amylose is likely confined to regions more accessible to enzymes than the more dense, less hydrated crystalline regions. It is not surprising therefore to find that structural studies locate amylose within the amorphous region of amylopectin (Figure 4 and Table 1). The long chains of amylose and super-long chains of amylopectin (both synthesized by GBSS) may give structural support to the amylopectin backbone within the amorphous regions.

## 10. Amylopectin Synthesis

Amylopectin is built from the hilum resulting from the initiation process by three major groups of enzymes, SSs, SBEs and DBEs, working in a regulated, coordinated manner (Figure 5). The three enzyme groups functionally, and in many cases, physically interact with each other. Within each enzyme class there are multiple isoforms with distinct biochemical properties, and these will be outlined in the sections below, with emphasis on their contributions to the backbone structure of amylopectin. The functions assigned to each of the isoforms from the three classes of enzymes are a result of analysis of mutants and biochemical analysis of purified enzymes or recombinant proteins. Therefore, we need to exercise caution in interpreting such data from individual enzymes and assigning a specific role for each in amylopectin biosynthesis, since the resulting structure is a product of many inter-dependent, and coordinated enzyme activities.

### 10.1. Starch Synthases

All SS isoforms are structurally related and share conserved functional domains [133]. Three of the six known SS isoforms are directly involved in amylopectin biosynthesis, producing α-(1,4)-linked glucan chains of varying chain lengths, which can be further elongated, branched (by SBEs) or debranched (DBEs). These SS isoforms are SSI, SSII and SSIII, and are involved in the elongation of successively longer (higher DP) α-(1,4)-linked glucan chains by the addition of glucosyl residues onto the non-reducing end of a pre-existing glucan chain, ADP-Glc serving as the glucosyl donor [189,190,191]. Unlike GBSS, all the SS isoforms involved in amylopectin biosynthesis have a distributive mode of action whereby the α-(1,4)-linked chain is extended by a single Glc per enzyme-substrate interaction. The potential donor chains for the SS isoforms may vary, from pre-formed α-glucans (MOS) produced by other SSs, DBEs releasing branch chains during amylopectin trimming, disproportionating enzyme (DPE), or SP.

SSI elongates the shortest length MOS/α-glucan chains of DP 6 to 7, producing an intermediate-sized α-glucan chain of DP 8 to 12 (precise chain lengths utilized and produced by different SS isoforms varies depending on botanical source, but the information given for SSs here is for maize endosperm and is generally representative of many plants studied). Studies with SSI from barley indicate the enzyme has no affinity for maltotriose or maltotetraose, indicating it utilizes pre-existing short MOS produced by other enzymes [192]. The intermediate chains of DP 8 to 12 produced by SSI are optimal substrates for further elongation by SSII isoforms which in turn produce longer chains of DP 12 to 30. In addition to its role in granule initiation and hilum formation, SSIII further elongates the long glucan chains of DP 12 to 30 produced by SSII to produce the longest linear chains in amylopectin of DP > 30.

SSI, SSII, and to a lesser extent SSIII are found tightly associated with the starch granules being resistant to extensive washing with detergents. It is not fully understood how granule associated proteins become associated with the granule, but in the case of some SS isoforms such as SSI, it has been suggested that their binding affinity for α-glucans increases as glucan chain length increases such that they become locked on the substrate [193]. The actions of other SS isoforms and SBEs may reduce the propensity of individual enzymes to become trapped in the granule during starch synthesis. In addition, the deposition of certain enzymes to become granule associated may be related to their physical interactions with SSII [194].

### 10.2. The Contribution of SS Isoforms to the Building Block-Backbone Model

The bulk of the amylopectin structure by mass is made up of building blocks who’s short α-glucan chains are extended to form short external segments. The positioning of these intermediate length chains is such that they form helices which contribute to the crystalline nature of the 9 nm repeat structure. SSI and SSII form, respectively, the short and intermediate length glucans which form the major part of the amylopectin structure, namely the building blocks and short external segments (elements that would be considered as part of the clusters in the Hizukuri and French models). Studies in Arabidopsis suggest a critical role for functional interactions between SSI and SBE (class II) in determining the characteristic polymodal chain length distribution found in plant starches [195], and SS and SBEs physically interact [196,197]. The building blocks and resultant short external segments subtend the long glucan backbone. The long chains forming the backbone are likely a result of SSIII activity [198]. Indeed, one proposed function for SSIII in amylopectin synthesis in other structural models is the provision of the long, cluster-interconnecting chains of the amylopectin backbone [198]. All of the aforementioned α-glucan chain groups are found in abundance in plant amylopectins and, together with analysis of various SS mutants (see [48,133,199]), suggest a crucial role for SSI, SSII and SSIII in building the building blocks/blocklets and backbone (Table 1). In addition, the backbone may be supported by the super-long chains formed by GBSS [28,117].

### 10.3. Starch Branching Enzymes

Branching enzymes catalyze an irreversible reaction which give rise to an α-(1,6)-linked branch linkage from a linear α-(1,4)-linked glucan chain via the hydrolytic cleavage of a α-(1,4) bond within the α-glucan chain. The reducing end of the released α-glucan chain is transferred to a C6 hydroxyl of either the original glucan chain (intra-chain transfer), or an adjacent or neighbouring α-glucan chain (inter-chain transfer) [200,201]. The factors determining whether inter- or intra-chain transfer occur are not fully understood. However, analysis of the SBE reaction in potato tuber suggest that relative concentrations of linear α-(1,4) chains (MOS) play a role. In particular, closely associated α-glucan chains, e.g., in double-helical configuration, promote the inter-chain transfer mechanism [201,202]. The branching reaction, and the characteristics of various branching enzymes has a profound influence on the resulting polyglucan structure [203].

In starch biosynthesis branching frequency is restricted to approximately 5%, much lower than that of other polyglucans such as glycogen (approximately 9%), and branch linkages are confined to the amorphous regions of the 9 nm repeat structure in amylopectin [11]. There are broadly two classes of SBEs in plants, based on conserved amino acid sequence relationships, SBEI and SBEII [204,205,206], the two evolving more than 200 million years ago, prior to the monocot-dicot divergence [207]. SBEI and SBEII are the products of separate genes [208] and have distinct biochemical properties suggestive of specific roles in the determination of amylopectin structure. The assigned roles for the different SBE classes is based upon genetic and biochemical evidence (for details see a review [209]). Generally, the two classes differ in their substrate specificity and the lengths of the α-glucan chains transferred. SBEI and SBEII classes show different minimum chain length requirements for branching; for SBEI it is approximately DP 15, and for SBEII it is approximately DP 12 [186,208]. SBEI shows a preference for amylose and transfers long chains (up to DP 30, with the majority being DP 10 to 13) [186,187], whereas SBEII isoforms show high catalytic activity with amylopectin and transfer relatively short (DP 6 to 14) α-glucan chains [186,210]. In cereals and grasses the SBEII class is further divided into tissue-specific isoforms, termed SBEIIa (found mainly in leaves) and SBEIIb (largely endosperm-specific), each products of separate genes [208,211,212] and different glucan length transfer properties [213]. If the in vitro characteristics of each of the SBE isoforms is retained in vivo, then they are likely involved in determining different aspects of amylopectin fine structure. In general, SBEI appears to be expressed more in storage tissues than leaves and other photosyntheic tissues, suggesting an important role in determining the structural properties of storage, as opposed to transient, starches. The loss of SBEI in cereals shows minor effects on amylopectin fine structure, suggesting some overlap of function between the different SBE isoforms [214,215,216]. Evidence supporting a role for SBEI in storage starch formation comes from maize mutants which show reduced germination (and therefore degradation of storage starch by α-amylases) when SBEI is absent [217]. Interestingly loss of SBEI in the green alga *Chlamydomonas reinhardtii* shows reduced transient starch degradation, suggesting that when it is expressed SBEI creates amylopectin structures more amenable to amylolysis [218]. Not all plants express SBEI, and plants such as Arabidopsis and Canola (*Brassica napus* L.) possess only SBEII class enzymes. In these oil storing plants starch is restricted to photosynthetic tissues and undergoes diurnal turnover [219].

### 10.4. The Contribution of SBE Isoforms to the Building Block-Backbone Model

We propose that the role of SBEI is the formation of long branch chains found in amylose and “super-long” chains of amylopectin, whereas its other contributions to amylopectin structure have minor impact (Table 1). Numerous studies indicate the importance of the SBEII class in determining amylopectin fine structure and influencing overall starch content in many plants [48,133,209]. This is perhaps not surprising given that the bulk of the amylopectin structure is made up of the building blocks (and inter-block segments), and that the branch linkages associated with these structures are likely a result of SBEII action (Table 1). In many plants, including the grasses and cereals SBEII accounts for most of the measurable SBE activity [220,221,222], and its loss leads to major alterations in amylopectin architecture. Different expression levels of the two SBEII isoforms (SBEIIa and SBEIIb) in cereals may account for some of the observed differences between storage and transient starches [211,220]. Loss of SBEII leads to amylopectin with reduced branching frequency, increased long chains (apparent amylose content) and reduced starch content [180,203,206,223,224,225]. Conversely, over-expression of SBEII leads to starches with low crystallinity, demonstrating the importance of coordinating the rates of α-glucan chain elongation (SS) and branching [226,227].

### 10.5. Starch Debranching Enzymes

Starch debranching enzymes (DBE) play a crucial role in determining the water-insoluble properties of amylopectin in the starch granules of green algae and higher plants through amylopectin trimming [12,228,229]. The debranching activity was first described by Hobson et al., and originally termed R-enzyme [95]. DBEs are structurally related members of the α-amylase super-family and hydrolyze the α-(1,6)-branch linkages formed by branching enzymes [230]. During the evolution of plants and green algae the catabolic function of some isoforms of DBE was recruited to become an essential component of the starch biosynthetic pathway. Hydrolysis of selected α-(1,6)-branch points is thought to lead to the clustering of remaining branches in amylopectin which promotes local α-glucan chain interactions and α-helix formation which are likely important for the formation of semi-crystalline amylopectin structures [165,215,231,232]. Two classes of DBE are found in plants, isoamylase-type (ISA) and pullulanase (limit dextrinase)-type (LDA). ISA-type DBEs debranch amylopectin and other polyglucans, whereas LDA in addition to acting on amylopectin can debranch the α-(1,6)-linkages in the fungal polymer pullulan [230]. The major function of LDA, and an ISA-type DBE isoform termed ISA3 is removal of α-(1,6)-branch linkages from chains released during starch degradation [233,234,235]. Any role LDA may play in defining amylopectin structure during synthesis is not clear, although it may be involved in debranching amylose chains in rice endosperm [236]. The precise structural nature of the substrates for these DBEs in vivo is not clear, and there appears to be functional overlap between the different isoforms [234,237,238]. During starch biosynthesis two ISA isoforms (ISA1 and ISA2) are involved in trimming exposed branches formed by SBEs leaving clustered branches which are presumably inaccessible to DBE or amylolytic action by α- and β-amylases. The role of DBEs in starch biosynthesis has been deduced largely from analysis of mutants, the most well-known being the *sugary1* mutant in sweetcorn varieties of maize which produces a water-soluble polyglucan structurally similar to glycogen called phytoglycogen [239,240]. Biochemical analysis of *Chlamydomonas* ISA1 supports a role for ISA in “*glucan trimming*” and the formation of water-insoluble polyglucan, as the enzyme shows a low affinity for tightly spaced branches, leading to the removal of the more open and accessible α-(1,6)-linkages which impede the branch clustering necessary for insoluble polyglucan accumulation [241,242]. The action of DBEs releases MOS chain fragments which may be utilized by GBSS for amylose synthesis or SP and D-enzyme which may control availability of MOS for granule initiation [160]. Unlike other key enzymes involved in amylopectin and amylose synthesis (SS and SBE isoforms), DBEs remain soluble in the plastid stroma as opposed to becoming physically associated with the growing water-insoluble starch granule.

### 10.6. The Contribution of DBEs to the Building Block-Backbone Model

**Figure 6:**
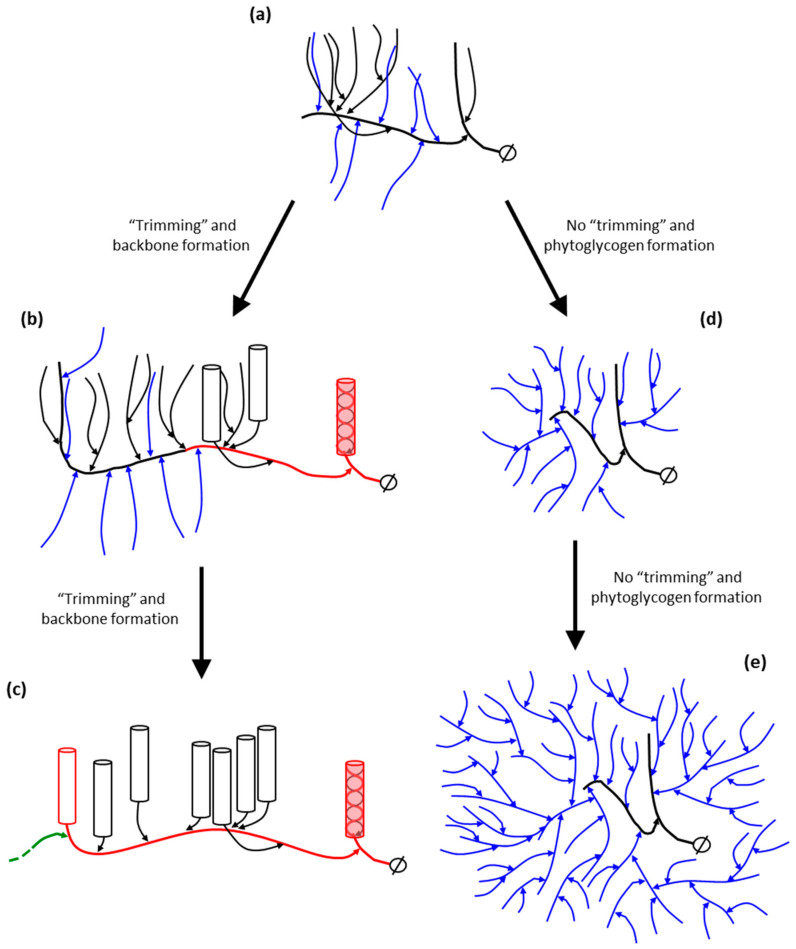
The trimming hypothesis of amylopectin adapted to the backbone model. (**a**) A “pre-amylopectin” is formed by the action of SSs and SBE, having a tightly branched structure. (**b**) The “pre-amylopectin” is “trimmed” through removal of chains (blue) by the action of DBE to form the backbone (red) with characteristic inter-block segments between the branched building blocks. The short chains that remain after the trimming form double-helices (cylinders). External chains of the long chains forming the backbone participate in double-helices (red cylinders). The backbone chain is elongated and extensively branched simultaneously with the trimming events. (**c**) The elongated structure is trimmed and the backbone continuous to be elongated eventually, with a new chain stub (green) introduced by SBEs. (**d**) An extensively branched molecule is formed without the trimming action, and (**e**) a water-souble phytoglycogen molecule is eventually the result of a continuous branching and chain elongation in the absence of DBE activity.

DBEs play a crucial, although indirect role in determining the fine structure of amylopectin. In relation to the cluster model of amylopectin structure described by Hizukuri and French amylopectin trimming was suggested to be necessary for the formation of clusters, which would have the optimal structure to form the crystallites [12]. However, within the context of the backbone model it is proposed that the DBE isoforms involved in amylopectin synthesis (ISA1, ISA2 and LDA) act on nascent building blocks and inter-block segments as they are synthesized at the periphery of the growing starch granule by SSs and SBEs and remove short external chains (see Table 1, and Figure 6).

As discussed in the model above, the major branched units in amylopectin are the building blocks, which are smaller (fewer branches) than the proposed clusters in the cluster model. With the backbone model, the function of the trimming, apart from prevention of phytoglycogen formation, is to prevent the formation of too large tightly branched entities (i.e., “clusters”), and to *allow the formation of inter-block segments* (Table 1), i.e., to allow the formation of a flexible backbone and helical segments along the backbone that brings the double-helices together to crystallize (cf. Figure 3). The possible events in this trimming are outlined in Figure 6. A part of the amylopectin molecule is originally formed with tightly positioned branches (Figure 6a). Subsequent trimming of this region by DBEs allows the further elongation of the emerging long chain in the growing backbone simultaneously with the double-helix formation of the remaining short chains in the trimmed section (Figure 6b). The elongated region is continuously trimmed and more double-helices are formed (Figure 6c), and so on. In the absence of trimming (lack of ISA1 activity) the extensively branched phytoglycogen is formed instead (Figure 6d,e), which is known to lack the fraction of long chains [243,244]. As mentioned above, Group 6 building blocks (with around 10 chains) are suggested to possibly represent remnants from an incomplete trimming of the backbone, as it is generally found in only small amounts in amylopectin [103].

### 10.7. Other Enzyme Activities

The following enzymes and enzyme groups are known to be an integral part of starch synthesis and metabolic pathway. However, despite knowledge of their immediate biochemical reactions their influence on final granule structure is not clear.

#### 10.7.1. α-Glucan Phosphorylation

The enzymes involved in covalent addition of phosphate moieties onto α-glucan chains of amylopectin (Figure 4 and Table 1) are indirectly responsible for causing localized modifications to amylopectin structure through changes in stability of chain-chain interactions [245]. The covalently-linked phosphate ([PO_4_]^3^^−^) residues on chains impart strong anionic charges which tend to repel neighboring phosphorylated oligosaccharides. It has been proposed that such repulsion opens and hydrates the α-glucan chains making them more susceptible to amylolytic attack [246]. In addition to affecting enzymes of starch turnover, phosphorylated α-glucans can influence the activities of starch biosynthetic enzymes and starch accumulation [247]. Phosphorylation of amylopectin is catalyzed by two plastidial dikinases, α-glucan water dikinase (GWD) and phospho-glucan water dikinase (PWD). GWD and PWD transfer the β-phosphate from ATP onto a glucosyl residue of an α-glucan chain [248]; GWD attaches phosphate at the C6 position, and PWD generally attaches phosphate to a pre-phosphorylated glucan at the C3 position (although pre-phosphorylation of the glucan substrate for PWD is not always an essential requirement) [246,248]. The agent(s) responsible for the deposition of starch phosphate at the C2 position is not known. The relative amounts of phosphate on starches of different botanical origins, and their relative distribution on the glucosyl residues on starch have been well documented (see earlier section). Despite this, the precise location and distribution of phosphate residues on α-glucan chains within the macromolecular structure of amylopectin is unclear (see Figure 4). Studies of phosphate distribution in different starches suggest an inverse relationship between amylopectin branching frequency and total phosphate content, with phosphate accumulating more readily on longer, less branched α-glucan chains [249]. It is not known how covalent addition of phosphate on amylopectin influences the higher order structures of the starch granule.

#### 10.7.2. α-Glucan Modification by DPE and SP

DPE and SP act on α-glucans and are implicated in the starch granule initiation pathway (see above). In addition, both enzymes have cytosolic counterparts which are likely involved in the turnover of transitory starch [133,250]. The chloroplast DPE also plays a role in facilitating nocturnal starch degradation [251]. However, there is also evidence that both enzymes play a role in starch synthesis by providing MOS and modifying their chain lengths; these modified glucans are then utilized by other biosynthesis enzymes. Both SP and DPE are found in the plastid stroma, and generally are not granule-associated, which is consistent with a role in soluble substrate modification/provision for other enzymes. Analysis of gene expression and measurements of enzyme catalytic activities in starch storing tissues is consistent with a role for both DPE and SP in starch biosynthesis [161,252,253,254,255]. SP is capable of α-glucan synthesis and degradation (phosphorolysis), and this equilibrium reaction is ostensibly controlled by the ratio of inorganic phosphate (Pi) to Glc1P. However, evidence suggests that the direction of the SP reaction is regulated beyond the availability of its substrates. In tissues with high Pi: Glc1P (which would be expected to promote phosphorolysis) such as rice endosperm, α-glucan synthesis predominates [256]. In *Chlamydomonas* the phosphorolytic reaction of SP is stimulated by DPE [257]. Hwang et al. [160] showed that DPE and SP form a functional protein complex in developing rice endosperm. The transglycosylation products (Glc and maltotriose) produced by DPE may be utilized by SP. During amylopectin biosynthesis the necessary trimming reactions catalyzed by DBEs release short chains (most of which are likely the short/intermediate chains from SBEII branching reactions) which may be modified by DPE, SP or the DPE/SP complex for modification by other enzymes (e.g., SSs). It is not known precisely how the reaction products of DBEs, DPE and SP are fed back into the starch biosynthesis pathway, or how these reactions are coordinated with SSs and SBEs.

### 10.8. Enzyme Complexes

Many of the enzyme classes involved in starch biosynthesis discussed in the above sections are coordinated and regulated in a variety of ways, from gene expression to various post-translational mechanisms including protein phosphorylation and redox modulation. The relationship between the formation of various protein complexes and amylopectin structure is unclear. However, many protein-protein interactions confer metabolic advantages, such as mediating substrate channeling [258], making it reasonable to assume that protein complexes involved in starch biosynthesis, such as the SS1/SSII/SBEIIb complex found in developing cereal endosperms [180,259] optimize construction of the building blocks more efficiently than the respective monomers. SSIII, on the other hand has been shown to be part of a large heteromeric protein complex [260,261]. Although SSIII is implicated in the construction of the long glucan chains which form the backbone of amylopectin, it may also act as a protein scaffold for other protein complexes [262]. Discussion of the many protein complexes involved in starch biosynthesis, and their regulation is beyond the scope of this review and can be found in a number of recent reviews [181,262,263].

## 11. Conclusions

The intention of this review is to integrate current knowledge of the pathway of starch biosynthesis and starch structure within the context of the *building block backbone* model as opposed to the more traditional cluster model. We argue that a holistic approach to understanding starch biosynthesis using structural analysis data to guide enzymology studies, and *vice versa*, mutually benefits both fields. Ultimately, we cannot make sense of the various enzymatic reactions and understand their roles without an accurate picture of the fine structure of the starch granule at the molecular, as well as higher order levels. In addition, we propose that both physical and biochemical analysis data of amylopectin fine structure are best described by the *building block backbone* model. We reviewed current knowledge of the many enzyme classes involved in starch biosynthesis with the intention of assigning their roles (where known) within the context of the structural motifs in the *building block backbone* model. Despite progress in many areas, important questions remain to be resolved. Beyond the initial linear structures produced during granule initiation we no nothing of the next immediate stages of granule formation involving branching, debranching and elongation. What limits granule size, particularly in storage starches? Despite an abundance of knowledge of enzymatic reactions and the smaller scale structures they produce (e.g., building blocks) we do not know the biological factors and/or self-assembly mechanisms responsible for higher order structures such as blocklets and growth rings. Most of the enzymes involved in amylopectin biosynthesis appear to operate within heteromeric protein complexes, and whilst discussion of these complexes is beyond the scope of this review, it is worth noting that we remain ignorant of the mechanisms by which these protein complexes define amylopectin structure.

## Figures and Tables

**Table 1:** Biological agents responsible for synthesis of fine structure components of starch.

Structural Feature	Function/Role	Enzyme/Proteins Involved
Hilum 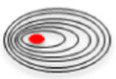	Initiation zone of granule growth facilitating formation of water-insoluble polyglucan	SSIII, SSIV, SSV, PTST2, PTST3, PII1/MRC (SP and DPE; provision of MOS)
Backbone 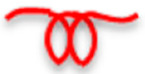	Linear α-(1,4)-linked glucan scaffolds from which building blocks are subtended	SSIII and/or SSIII protein complexes
Building Blocks 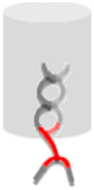 Short External Segments 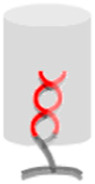	Major structural component of amylopectin. The framework from which short/intermediate α-glucan chains are produced which form water-excluding helical coils. Major structure responsible for the 9 nm repeat observed in coiled stacks of building blocks (blocklets?)	SSI, SSII, SBEII. The building block results from the combined actions of SS and SBE, likely SSI and/or SSII and SBEII isoforms. Further extension of the chains into short external segments is likely catalyzed by SSII. SSI, SSII and SBEII form protein complexes whose catalytic activity may be responsible for these building blocks and the subsequent short/intermediate chains pairs of which form the helical coils shown as cylinders. (DBE activity allows short/intermediate chains arising from building blocks to form water-insoluble structures via removal of selected α-(1,6)-branch linkages)
Interblock Segments 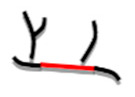	Provide spacing between building blocks. Such spacing may influence larger scale packing of groups of building blocks possibly determining A- or B-type structural allomorphs as viewed by X-ray scattering.	SSIII and/or SSIII protein complexes (DBEactivity to reduce excess branching between building blocks)
Superlong Chains 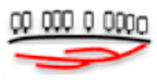	Packing within amorphous regions. Interacting with structurally similar amylose chains and providing structural stability.	GBSS and/or SSIII
Linear amylose 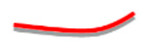 Branched amylose 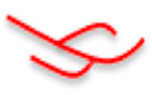	Unknown. Possibly aids packing glucan within amylopectin matrix in less dense amorphous regions.	GBSS (I and II) and PTST1. SBE isoform responsible for branching amylose unknown, possibly SBEI (SP, DPE, DBE; provision of MOS for GBSS)
Phosphate Groups 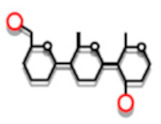	Anionic phosphate groups cause localized mutual repulsion of glucan chains leading to structural instability, making structure prone to amylolytic action.	GWD; adds phosphate group at C6 of glucosyl residue on α-(1,4)-linked chain PWD; adds phosphate group at C3 of phosphoglucan Unknown activity responsible for phosphorylation at C2
